# Epinephrine Use during Newborn Resuscitation

**DOI:** 10.3389/fped.2017.00097

**Published:** 2017-05-01

**Authors:** Vishal S. Kapadia, Myra H. Wyckoff

**Affiliations:** ^1^Pediatrics, University of Texas Southwestern Medical Center, Dallas, TX, USA

**Keywords:** epinephrine, neonatal resuscitation, asphyxia, newborn, delivery room, infants

## Abstract

Epinephrine use in the delivery room for resuscitation of the newborn is associated with significant morbidity and mortality. Evidence for optimal dose, timing, and route of administration of epinephrine during neonatal resuscitation comes largely from extrapolated adult or animal literature. In this review, we provide the current recommendations for use of epinephrine during neonatal resuscitation and also the evidence behind these recommendations. In addition, we review the current proposed mechanism of action of epinephrine during neonatal resuscitation, review its adverse effects, and identify gaps in knowledge requiring urgent research.

## Introduction

Approximately 10% of newborns require some assistance to begin breathing at birth (1). Majority of these newborns improve without the need for cardiac compression or epinephrine if skillful positive-pressure ventilation is initiated in a timely manner. Less than 0.1% of all newborns require epinephrine, making epinephrine use in delivery room neonatal resuscitation an uncommon event (2, 3). Newborns who do require extensive cardiopulmonary resuscitation (CPR) including epinephrine have a high incidence of mortality. Those who survive frequently suffer from poor long-term neurodevelopmental outcomes (4–7).

The majority of recommendations regarding indication, dose, and route of administration of epinephrine in the delivery room are based on extrapolations from adult and animal studies. The infrequent use of epinephrine in the delivery room and ethical dilemmas in designing a clinical trial for examining the role of epinephrine during neonatal resuscitations make it very difficult to obtain high levels of evidence for recommendations regarding epinephrine use during neonatal resuscitation. Many of the animal and adult data come from a non-perfusion ventricular fibrillation arrest, which is not the pathophysiology of a newborn in the delivery room who suffers from an asphyxial arrest. Another major limitation of extrapolation from these studies is that newborns in the delivery room have unique transitional physiology including fluid-filled alveoli, an open ductus arteriosus, and high pulmonary pressures with limited pulmonary blood flow. Newly born infants must transition from fetal to newborn circulation. In the era of evidence-based medicine, due to lack of rigorous scientific evidence, proper use of epinephrine including dose and route of administration remains controversial. Even though epinephrine is not commonly needed in neonatal resuscitation, its association with death and poor prognosis raises questions as to whether optimization of epinephrine use and dosing, specifically tailored to the unique circumstances of the newly born infant, could improve outcomes.

This review aims to describe current recommendations for epinephrine use in neonatal resuscitation, the evidence behind such recommendations, and the critical knowledge gaps.

## History of Epinephrine Use in Neonatal Resuscitation

Epinephrine is the only medication recommended during neonatal resuscitation in the delivery room (8, 9). Naloxone, sodium bicarbonate, and other vasopressors are currently not considered a part of acute resuscitation but can be used postresuscitation for special circumstances (9–11).

Management of the airway and assisted ventilation of the newborn baby can be found in ancient texts dating back to the Old Testament of the Bible, the Talmud, and Hippocrates (12, 13). However, reports of medication use in neonatal resuscitation can only be found after the early 1950s with the evolution of modern neonatology (13, 14). George Oliver and Edward Schaffer in 1893 first showed that adrenal glands contained a substance with distinct pharmacological properties (14, 15). It is a naturally occurring catecholamine produced by chromaffin cells at the adrenal medulla and stored in chromaffin granules. In 1897, John Abel in the United States prepared crude adrenal extracts and called them epinephrine (16). Epinephrine was used first time in pulseless patients in around 1906 by Crile and Dolley (17). Its resuscitative properties were further investigated by Wiggers in the 1930s and Redding and Pearson in the 1960s (18, 19).

## Hemodynamic Effects of Epinephrine

Epinephrine stimulates all four adrenergic receptors (α_1_, α_2_, β_1_, and ß_2_) *in vivo*. When looked at in isolation, stimulation of the different adrenergic receptors by epinephrine results in different and sometimes opposing effects. It causes peripheral vasoconstriction *via* stimulation of α_1_ receptors in vascular smooth muscle cells. By stimulating β1 receptors in the myocardium, it causes chronotropy (increased heart rate), inotropy (increased contractility), dromotropy (increase conduction velocity), and lusitropy (increased rate of myocardial relaxation) (10, 20–22). Stimulation of α_2_ receptors leads to presynaptic inhibition of nor-epinephrine release in the central nervous system and vasoconstriction of coronary arteries. Through β_2_ receptor stimulation, it causes vascular smooth muscle relaxation and increased myocardial contractility, but these effects are usually minor. *In vivo* effects of epinephrine depend on the dose of epinephrine, number of receptors available on target tissues, the affinity of these receptors, and local target tissue environments (23).

## Mechanism of Action During CPR

Initially it was believed that epinephrine causes return of spontaneous circulation (ROSC) in cardiac arrest *via* its myocardial stimulant effects (β adrenergic effects: chronotropic and inotropic) (10). In the 1960s, Redding demonstrated in dogs that the pure α-agonist, methoxamine, was as effective as epinephrine in achieving ROSC during CPR, whereas the pure β-agonist, isoproterenol, was no more effective than CPR alone (19). Otto et al. who used pretreatment with α-adrenergic blockade (phenoxybenzamine) and β-adrenergic blockade (propranolol) before infusing epinephrine confirmed that α-adrenergic stimulation is the most important action of epinephrine for ROSC in CPR (24).

**Figure 1:**
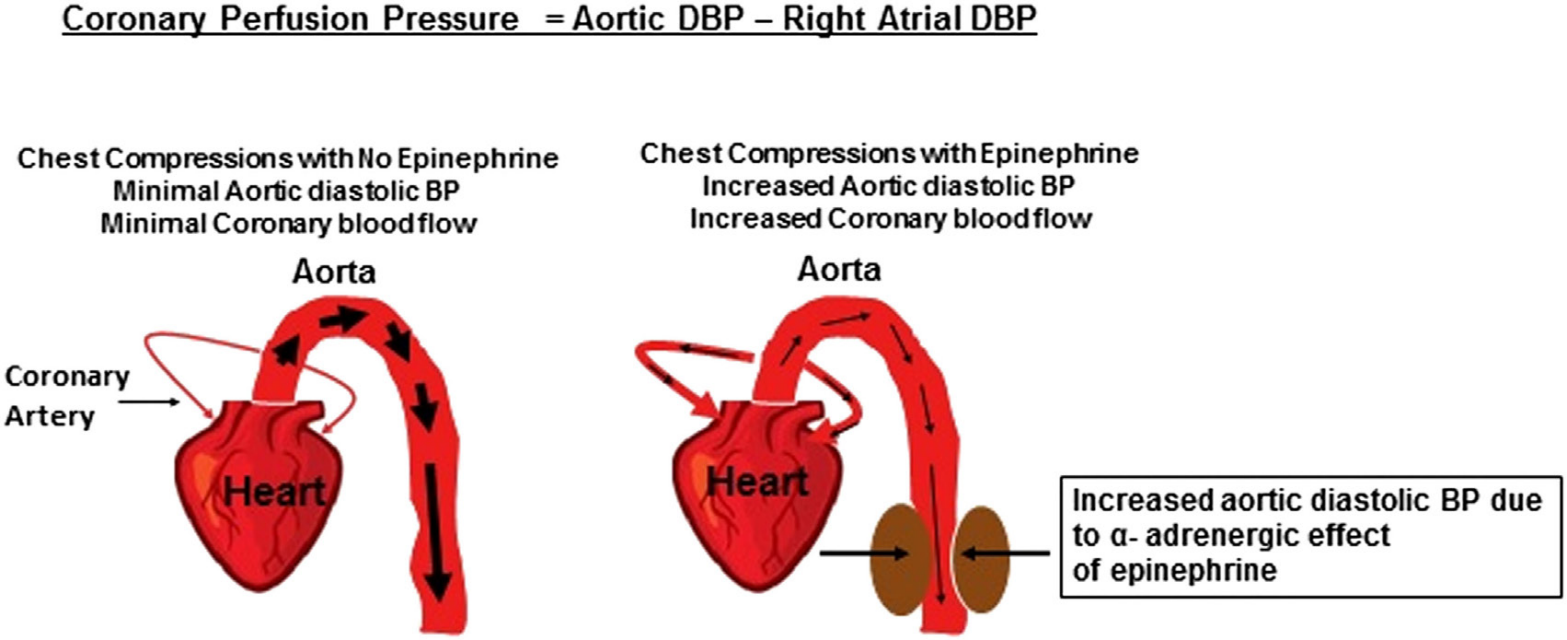
**Epinephrine and coronary perfusion pressure**.

It is now established that the most reliable method for determining the effectiveness of CPR is to measure aortic diastolic blood pressure or coronary perfusion pressure (25). When heart muscles do not receive adequate blood flow and/or oxygen, their energy substrate is depleted. In turn, heart muscles stop contracting and the heart stops pumping. To restart the cardiac pump, it is critical that myocardial perfusion with oxygenated blood is reestablished. In acidotic asphyxiated neonates, there is loss of peripheral vascular tone, i.e., maximum vasodilation. When chest compressions are performed, blood from the cardiac chambers takes the path of least resistance and thus preferentially flows through aorta and into peripheral circulation rather than into narrow more constricted coronary arteries that have high resistance (Figure 1). The use of epinephrine in this situation results in intense peripheral vasoconstriction. This elevates the aortic to right atrial pressure gradient during the relaxation phase of CPR (26–29). Due to this pressure gradient, blood during chest compressions enters the coronary arteries and myocardial blood flow increases. Hence, this pressure gradient is called the coronary perfusion pressure. As oxygenated blood enters the coronary circulation, it facilitates resynthesis of adenosine triphosphate within myocardial mitochondria improving myocardial contractility and viability. In animal models and humans, coronary perfusion pressure correlates directly with myocardial blood flow, which is a good predictor of ROSC.

Although minor, β2 receptor-mediated coronary vasodilation may contribute to improved coronary perfusion following epinephrine administration (10, 21, 30). Cerebral electrographic activity and cerebral oxygen uptake improves following epinephrine administration during CPR as cerebral blood flow increases due to epinephrine-induced peripheral vasoconstriction (28, 29). Through its α receptor stimulation, epinephrine may counteract carotid artery collapse induced by elevated intrathoracic pressures due to CPR and further optimize blood flow (28).

Studies utilizing posttransition asphyxia animal model have demonstrated the importance of epinephrine, where after asphyxia cardiac arrest, chest compressions alone were ineffective, but majority of animals reached the critical diastolic blood pressure (rising aortic to right atrial pressure gradient) and ROSC after epinephrine administration (31–33). It is important to note that these studies also showed that interruptions in chest compression lead to lowering of diastolic blood pressure, thus highlighting the importance of minimizing interruptions in cardiac compressions during CPR (31–33).

The majority of the above information was obtained from adult animal studies, posttransitioned neonatal animal studies, or human adult studies. No studies in term or preterm newborns or animal models with newborn transition physiology have investigated the mechanism of action of epinephrine during CPR. The distribution and maturation of α and β receptors in term and preterm newborns remain unknown (23).

## Current Indication for Epinephrine During Neonatal CPR

Bradycardia in newly born infants is usually the result of inadequate aeration of lungs and ventilation or profound hypoxemia and acidosis from prior poor placental perfusion. Hence, effective ventilation is the top priority during delivery room resuscitation of the bradycardic newborn. Current resuscitation guidelines recommend that epinephrine should be used if the newborn remains bradycardic with heart rate <60 bpm after 30 s of what appears to be effective ventilation with chest rise, followed by 30 s of coordinated chest compressions and ventilations (1, 8, 9).

## Optimal Dose and Route of Administration of Epinephrine During CPR

Epinephrine during neonatal CPR in the delivery room can be given by three routes: intravenous, endotracheal (ET), and intraosseous (Table 1).

**Table 1:** **Epinephrine use during newborn resuscitation: route, dose, and summary of evidence**.

Route	Dose	Summary of evidence
Intravenous	0.01–0.03 mg/kg	Preferred route and appear to be more efficacious than other routes Dose extrapolated from adult experience High-dose epinephrine offers no advantage and is associated with increased postresuscitation adverse effects and increased mortality Dose escalation studies in neonatal animal model with transition physiology are urgently needed
Endotracheal (ET)	0.05–0.1 mg/kg	Less effective than IV route Achieved plasma concentration is less and it peaks slower with ET epinephrine compared to IV epinephrine Can be used until IV access is available
Intraosseous	0.01–0.03 mg/kg	Limited evidence compared to IV route Providers frequently involved in newborn resuscitation feel more comfortable with rapid UVC insertion compared to IO route
Intramuscular	Not recommended	Very limited evidence Significant tissue damage at local site

### Intravenous Epinephrine

This is the preferred route of administration during neonatal CPR in the delivery room as it appears to be more efficacious compared to other routes (1, 8, 9). The umbilical vein is a rapidly accessible, direct intravenous route. If epinephrine use is anticipated based on risk factors and no response to optimized positive-pressure ventilation (preferably *via* a secured airway), one team member should prepare to place an umbilical venous catheter, while the others continue to provide ventilation and chest compression. Chest compressions should be provided from head of the bed to allow adequate access to place the umbilical venous catheter (1).

The optimal dose of intravenous epinephrine has been the subject of much debate. In animal ventricular fibrillation models, Redding and Pearson demonstrated that intravenous epinephrine of 1 mg (0.1 mg/kg in 10 kg dogs) increased ROSC when combined with ventilation and chest compressions alone (19). Human studies following this study did not take into account the weight difference between the 10-kg dogs that were studied and the average adult weight, which is 7- to 10-fold more. Surprisingly, even with such low doses, epinephrine was reported to be effective in achieving ROSC in adult CPR (34). As there are no neonatal epinephrine dosing studies, the recommended dose was extrapolated from the adult experience with a suggested dosing range of 0.01–0.03 mg/kg. Given the overlooked weight difference between dogs in the study by Redding and Pearson (19) and humans, studies were conducted to see if higher dose epinephrine would be more efficacious. Initially, studies in ventricular fibrillation adult animal model showed increased ROSC and improved cerebral and coronary blood flow with escalating doses of epinephrine (35). Based on these data, adult and pediatric resuscitation guidelines started recommending using 0.1 mg/kg high dose of epinephrine if no response was seen with standard dose epinephrine (36). Clinical studies conducted later found that high-dose epinephrine (0.1 mg/kg) is not more effective and may be harmful (35, 37, 38).

#### Animal Data

Berg et al. in a pediatric asphyxia swine model demonstrated that high-dose epinephrine did not result in increased ROSC, and in fact, there was higher postresuscitation mortality (39). Burchfield et al. in a neonatal lamb model demonstrated that high-dose epinephrine reduced stroke volume and cardiac output (40). McCaul et al. demonstrated dose-related adverse outcomes with higher tachycardia, hypertension, mortality, and increased troponin with high-dose epinephrine in a rat model (41). Observation of hypertension following hypotension with high-dose epinephrine is especially important for preterm newborns who are vulnerable to development of intraventricular hemorrhage with fluctuations in blood pressure (42, 43).

#### Adult Data

Meta-analysis of randomized control trials in adult cardiac arrest patients demonstrated increased ROSC with high-dose epinephrine but no improvement in survival to hospital discharge (35).

#### Older Children

Perondi et al. randomized 68 children (mean age of 6 years) to either 0.1 versus 0.01 mg/kg for the second dose of epinephrine after failure of standard first dose (0.01 mg/kg) (38). This study demonstrated that ROSC rates were similar between both groups. Alarmingly, no child survived in the high-dose epinephrine group compared to 21% survival in the standard epinephrine group. Patterson et al. confirmed these findings that high-dose epinephrine did not confer any benefits but reduced survival when arrest was precipitated by asphyxia (37).

#### Neonatal Data

There is a stark absence of any neonatal studies including randomized controlled trials studying any dose of epinephrine. Halling et al. described in an observational study of 20% success rate with single standard dose of IV epinephrine. Multiple doses were needed by large number of newborns (3).

In summary, these data suggest that there is no advantage with high-dose epinephrine, and it is associated with postresuscitation hypertension, tachycardia, and increased mortality especially following cardiac arrest from asphyxia. Neonatal data remain sparse, and dose escalation studies in appropriate neonatal models with transition physiology are urgently needed.

### ET Epinephrine

Although the ET route is readily available and less time consuming than establishing an intravenous or intraosseous access, it appears to be less effective (36, 44, 45). However, until intravenous access is available, some clinicians may choose to give epinephrine ET (1, 9). Currently, the recommended dose is 0.05–0.1 mg/kg, which is much higher than the recommended intravenous epinephrine dose (1, 9).

#### Adult Animal Data

Redding et al. were the first to suggest the use of ET epinephrine during cardiac arrest (46). In a ventricular fibrillation pig model, Crespo et al. compared 0.01 versus 0.1 mg/kg ET epinephrine doses (47). The study demonstrated that higher dose was able to achieve higher plasma concentrations of the drug but that did not translate to higher blood pressure. Roberts et al. also investigated different ET epinephrine doses and compared them with equivalent intravenous epinephrine doses (48). The study demonstrated that the peak concentration of epinephrine was found in 15 s after either route of administration, but with ET epinephrine, blood concentrations were more sustained. Importantly maximum plasma concentration achieved by ET epinephrine was one-tenth of the plasma concentration achieved by the intravenous route. Vali and Lakshminrusimha conducted a study of ET versus intravenous epinephrine in a fetal lamb model of asphyxia where animals had not yet transitioned to newborn circulation (49). They demonstrated that plasma epinephrine peaks much faster and higher compared to ET epinephrine although no difference in rates of ROSC was observed between either group.

#### Human Adult Data

Many retrospective adult case series have noted ET epinephrine to be less effective than IV epinephrine in achieving ROSC during CPR (36, 44, 45).

#### Neonatal Data

Four case series in neonates noted some evidence of absorption or cardiovascular improvement following ET epinephrine administration, but doses were 10 times higher than typical intravenous doses, and the majority of newborns had bradycardia, not asystole (50–52). Barber and Wyckoff reported on a retrospective review of all neonates who received epinephrine in the delivery room during the study period (2). The study demonstrated that the majority of infants received their first dose as ET epinephrine. They found that ET epinephrine dose of 0.01–0.03 mg/kg failed to re-establish HR > 60 bpm two-thirds of time. In the neonates who failed to respond to ET epinephrine, 77% of them responded to subsequent intravenous epinephrine. ET epinephrine efficacy may be limited in the newly born due to dilution by non-mobilized lung fluid. Elevated pulmonary arterial pressure in the presence of patent ductus arteriosus could result in right-sided cardiac output bypassing the lungs and thus limiting epinephrine absorption from the lung (23, 25). Based on this evidence, guidelines recommended an increase in ET epinephrine dosing from 0.01 to 0.03 to 0.05 to 0.1 mg/kg (1, 9). Halling et al. presented a retrospective review comparing the dosing from 0.03 to 0.05 mg/kg (3). They found no improvement in rates or time of ROSC with the higher ET epinephrine dose. It is possible that there may not be an optimal ET epinephrine dose. Current guidelines stress the importance of education, practice, and preparation to rapidly establish IV access in delivery room for newborns who need epinephrine during delivery room resuscitation (9).

### Intraosseous Epinephrine

Simulation studies have shown that for inexperienced personnel, establishment of an intraosseous line was faster and easier than the placement of umbilical catheters (53). In a neonatal case series of 27 neonates who received intraosseous epinephrine for resuscitation, no short-term complications were demonstrated (54). Also many critical clinical outcomes were not described. Given the comfort level that can be achieved by neonatal providers for rapid placement of umbilical catheters and limited evidence regarding IO placement in delivery room, IV epinephrine is preferred (1).

### Intramuscular Epinephrine

Mauch et al. demonstrated that 0.1 mg/kg of IM epinephrine resulted in similar ROSC and survival in infant piglet cardiac arrest model (55). Case reports indicate that intramuscular epinephrine of 0.02 mg/kg causes significant tissue damage at injection site (56). Currently, intramuscular epinephrine is not recommended for neonatal CPR.

## Adverse Effects of Use of Epinephrine During CPR

Epinephrine especially with repeated doses or with high doses can cause postresuscitation hypertension and tachycardia (39, 57). This can result in injury to various organ systems especially in preterm neonates. Excess epinephrine due to its vasoconstrictive properties can impair blood flow to various organs such as kidneys and intestines. Epinephrine can also result in elevation of pulmonary arterial pressures and increase myocardial oxygen consumption and demand through its β adrenergic effects (58, 59). This may be detrimental especially in situations where hypoxia persists and oxygen delivery is impaired. It has also been associated with imbalance of various neurotransmitters such as gamma-aminobutyric acid, dopamine, serotonin, acetylcholine (60–63). It can impair blood–brain barrier and possibly decrease the threshold for seizures (62, 64).

## Alternatives to Epinephrine in DR

Given the limitations of epinephrine in neonatal CPR, there is a great interest in finding other vasoconstrictors that have fewer detrimental side effects. Vasopressin has been studied in the adult literature as an alternative. Endogenous vasopressin levels were found to be higher in successfully resuscitated adults compared to those who died. Vasopressin through V1 receptors is a potent vasoconstrictor of blood vessels in the skin, skeletal muscle, and mesenteric blood vessels (10, 65, 66). It does not have any stimulant effect on the myocardium, and at low doses, it can vasodilate coronary, pulmonary, and cerebral vessels. Even though it has these theoretical benefits over epinephrine, in randomized control trials in adults, vasopressin has not found to be more effective than epinephrine (67). A cohort study on pediatric in-hospital cardiac arrest vasopressin was found to be less effective and associated with higher mortality (68). In neonatal piglet posttransition asphyxia model, McNamara et al. showed that vasopressin resulted in improved survival, lower postresuscitation troponin, and less hemodynamic compromise compared to epinephrine (69). No human neonatal data exist regarding vasopressin in CPR. Studies with neonatal animal models with transition physiology are urgently needed.

## Other Considerations for Epinephrine in the Delivery Room

### Interval between Doses

The current recommendation is to repeat the dose of IV epinephrine every 3–5 min if the heart rate remains less than 60 bpm (1, 9). Vali and Lakshminrusimha in a fetal lamb asphyxia model demonstrated an incremental increase in plasma epinephrine concentration with repeated IV epinephrine doses every 3–5 min (49). Warren et al. performed retrospective review of in-hospital cardiac arrest in adults and found the optimal interval to repeat dose to be 9–10 min instead of 3–5 min (70). Linner et al. gave epinephrine before chest compressions to bradycardic and severely asphyxiated newborn piglets and demonstrated that this strategy did not improve ROSC or cerebral circulation (71). More studies are needed to find out optimal interval between doses, but current evidence would suggest that more frequent or early epinephrine does not seem to be more beneficial.

### Flush Volume after IV Epinephrine Dose through Low UVC

Currently recommended flush volume after IV epinephrine dose is 0.5–1 ml (1). Vali and Lakshminrusimha showed higher incidence of ROSC and faster ROSC with right atrial epinephrine compared to low UVC epinephrine in fetal lamb asphyxia model (49). It is possible that the currently recommended flush volume will deposit the epinephrine in umbilical vein but might not be enough to reach the heart. It is unclear if current flush volume is adequate and if higher flush volume may result in faster rise and higher epinephrine plasma concentrations. Studies are underway to answer this question.

## Outcomes in Newborns Who Require Epinephrine in the Delivery Room

Cohort study data suggest that epinephrine is needed in <0.1% of all liver born deliveries (2, 3) although there is a large variation among different centers. Severe fetal acidemia, malpositioned ET tubes, and ineffective ventilator support contribute to the higher use of delivery room epinephrine (72, 73). Thus, it remains critical that neonatal providers focus on optimizing positive-pressure ventilation including placement of an alternate airway as a part of their ventilation corrective measures if a newborn is not responding to initial positive-pressure ventilation. Provision of effective ventilation that moves the chest should eliminate or reduce unnecessary intensive CPR. Term infants who require intensive CPR including multiple epinephrine doses and those whose Apgar score remain low at 10 min of life suffer from high incidence of death or poor neurodevelopmental outcomes (4, 5). In preterm infants due to lack of good evidence for use of epinephrine and its adverse effects of epinephrine especially postresuscitation hypertension, outcome data become even more important. Multiple retrospective observational studies have noted that preterm neonates requiring CPR and epinephrine have significantly lower survival, higher incidence of early onset sepsis, NEC, grade 3–4 intraventricular hemorrhage, cystic periventricular leukomalacia, bronchopulmonary dysplasia, and neurodevelopmental impairment (7, 74–76). These studies frequently suffer from small numbers and selection bias as the most compromised and sicker preterm neonates may require CPR but all studies point toward worse outcomes associated with extensive delivery room CPR. These data suggest that optimization of CPR and epinephrine use in delivery room has potential to impact outcomes significantly.

## Conclusion

Epinephrine use in delivery room remains uncommon especially when neonatal providers focus on effective positive-pressure ventilation. Epinephrine use in delivery room is associated with high mortality and poor long-term outcomes. Recommendations regarding epinephrine use including dose and route are based mostly on extrapolation of data from animals or adult literature. Even the majority of available animal data come from ventricular fibrillation cardiac arrest models and posttransition models that have little in common with newborns in the delivery room. There is a scarcity of human neonatal term and preterm epinephrine data even in the form of observational studies. Based on the limited available literature, intravenous epinephrine is preferred to ET epinephrine. Clinical and animal studies in transition neonatal models are urgently needed to identify optimal indication, timing, dose, route, and alternatives to epinephrine in neonatal CPR.

## Author Contributions

VK performed the literature review, created first draft of the article, revised the draft, and created and approved the final draft of the article. MW critically reviewed the first draft, revised the draft, approved the final draft of the article, and contributed substantially to this manuscript.

## Conflict of Interest Statement

The authors declare that the research was conducted in the absence of any commercial or financial relationships that could be construed as a potential conflict of interest.
