# Corrigendum: Epinephrine Use During Newborn Resuscitation

**DOI:** 10.3389/fped.2018.00399

**Published:** 2018-12-18

**Authors:** Vishal S. Kapadia, Myra H. Wyckoff

**Affiliations:** Pediatrics, University of Texas Southwestern Medical Center, Dallas, TX, United States

**Keywords:** epinephrine, neonatal resuscitation, asphyxia, newborn, delivery room, infants

In the original article, there was a typographical error in Table 1, as published. The endotracheal (ET) epinephrine dose should be “0.05–0.1 mg/kg.” In Table 1, it was mistakenly written as “0.05–1 mg/kg.” The corrected Table 1 appears below.

**Table 1:** Epinephrine use during newborn resuscitation: route, dose, and summary of evidence.

**Route**	**Dose**	**Summary of evidence**
Intravenous	0.01–0.03 mg/kg	Preferred route and appear to be more efficacious than other routes Dose extrapolated from adult experience High dose epinephrine offers no advantage and is associated with increased post-resuscitation adverse effects and increased mortality Dose escalation studies in neonatal animal model with transition physiology are urgently needed
Endotracheal (ET)	0.05–0.1 mg/kg	Less effective than IV route Achieved plasma concentration is less and it peaks slower with ET epinephrine compared to IV epinephrine Can be used until IV access is available
Intraosseous	0.01–0.03 mg/kg	Limited evidence compared to IV route Providers frequently involved in newborn resuscitation feel more comfortable with rapid UVC insertion compared to IO route
Intramuscular	Not recommended	Very limited evidence Significant tissue damage at local site

The authors apologize for this error and state that this does not change the scientific conclusions of the article in any way. The original article has been updated.

